# Salicylate of Lithia in Rheumatism

**Published:** 1886-02

**Authors:** 


### Salicylate of Lithia in Rheumatism.

M. Vulpian has presented, before the Acadêmie de Mêdecine, the following summary of the result of his researches upon the use of salicylate of lithia in rheumatism :

In acute articular rheumatism and in acute attacks of gout, the salicylate of lithia appears to be as efficacious as the similar sodium salt. The lithia salt is occasionally useful in reducing the still slightly painful joints left after treatment with the sodium salt.

The forms of acute rheumatism in which the fibrous tissue is especially attacked, respond more readily to the lithia salt, which is also the more effective in subacute progressive articular rheumatism. By the use of the salt last named, there has been obtained a noteworthy abatement of the symptoms of chronic articular rheumatism.

The maximum efficient dose of salicylate of lithia is seventy-five grains daily, while sixty grains daily are generally sufficient. Its usual physiological effects are, more or less pronounced headache, vertigo, and considerable deafness, but the buzzing and whistling and other subjective disturbances of hearing, so common after the use of the salicylate of soda, are here comparatively rare.—*L' Union Mêdicale*, Dec. 10, 1885.

